# Leg-length inequality is not associated with greater trochanteric pain syndrome

**DOI:** 10.1186/ar2433

**Published:** 2008-05-29

**Authors:** Neil A Segal, William Harvey, David T Felson, Mei Yang, James C Torner, Jeffrey R Curtis, Michael C Nevitt, the Multicenter Osteoarthritis Study Group

**Affiliations:** 1 Department of Orthopedics & Rehabilitation, University of Iowa Hospitals and Clinics, 200 Hawkins Drive, 0728 JPP, Iowa City, IA 52242-1088, USA; 2 Boston University Clinical Epidemiology Research Training Unit, Boston University School of Medicine, 715 Albany Street, A203, Boston, MA 02118, USA; 3 Department of Epidemiology, University of Iowa School of Public Health, C-21P-1 GH, Iowa City, IA 52242, USA; 4 Division of Clinical Immunology and Rheumatology, University of Alabama at Birmingham, 510 20th Street South, FOT 840, Birmingham, AL 35294, USA; 5 Department of Epidemiology & Biostatistics, University of California, San Francisco, Box 0560, 185 Berry Street, Lobby 4, Suite 5700, San Francisco, CA 94107-1762, USA

## Abstract

**Introduction:**

Greater trochanteric pain syndrome (GTPS) is a common condition, the pathogenesis of which is incompletely understood. Although leg-length inequality has been suggested as a potential risk factor for GTPS, this widely held assumption has not been tested.

**Methods:**

A cross-sectional analysis of greater trochanteric tenderness to palpation was performed in subjects with complaints of hip pain and no signs of hip osteoarthritis or generalized myofascial tenderness. Subjects were recruited from one clinical center of the Multicenter Osteoarthritis Study, a multicenter population-based study of community-dwelling adults aged 50 to 79 years. Diagnosis of GTPS was based on a standardized physical examination performed by trained examiners, and technicians measured leg length on full-limb anteroposterior radiographs.

**Results:**

A total of 1,482 subjects were eligible for analysis of GTPS and leg length. Subjects' mean ± standard deviation age was 62.4 ± 8.2 years, and 59.8% were female. A total of 372 lower limbs from 271 subjects met the definition for having GTPS. Leg-length inequality (difference ≥ 1 cm) was present in 37 subjects with GTPS and in 163 subjects without GTPS (*P *= 0.86). Using a variety of definitions of leg-length inequality, including categorical and continuous measures, there was no association of this parameter with the occurrence of GTPS (for example, for ≥ 1 cm leg-length inequality, odds ratio = 1.17 (95% confidence interval = 0.79 to 1.73)). In adjusted analyses, female sex was significantly associated with the presence of GTPS, with an adjusted odds ratio of 3.04 (95% confidence interval = 2.07 to 4.47).

**Conclusion:**

The present study found no evidence to support an association between leg-length inequality and greater trochanteric pain syndrome.

## Introduction

In 2001, 33% of US adults reported having had pain, aching, stiffness or swelling around a joint on most days for at least one of the past 12 months [1]. Since more than 70 million adults in the United States have chronic musculoskeletal complaints, a better understanding of treatable, nonarthritic musculoskeletal conditions would inform therapy to reduce morbidity.

Greater trochanteric pain syndrome (GTPS) is a common but frequently overlooked condition that may present in the context of hip or spine pain or pathology. The anatomic relationships between three bursae (subgluteus maximus, medius, and minimus), the hip abductor/external rotator muscles, the greater trochanter, and the overlying iliotibial tract may predispose this area to biomechanical irritation. Although abnormal biomechanics of the lower limb are frequently cited as predisposing factors for GTPS, however, few have been well studied [2].

Some musculoskeletal textbooks suggest that leg-length inequality (LLI) contributes to trochanteric pain by adding stress to the area [3,4]. These references suggest that GTPS tends to occur on the longer leg because of the increased stress imposed on the abductor muscles and fascia lata in the context of pelvic obliquity [4,5]. Although these purported associations are theoretically plausible, they have not been evaluated. We could find only one report of an association between GTPS and LLI listed on MEDLINE. The report detailed four patients with severe hip osteoarthritis in which GTPS was present on the *shorter*, rather than longer, lower limb [6]. Earlier case series also recommended a heel lift on the affected side for mild GTPS, due to a belief that GTPS occurs on the shorter lower limb [7].

Despite the lack of published evidence regarding LLI and GTPS, there is a sound biomechanical basis for hypothesizing that a relationship could exist. A limb-length inequality >2 cm has been reported to alter gait mechanics, even with compensatory mechanisms [8-10]. Differences as small as 1.2 cm have been reported to predict hip osteoarthritis [11,12], and even differences <1 cm have been found in patients with chronic low back pain [13,14]. Gait analysis has also revealed that moments are significantly greater throughout the longer limb [15], and there is greater pelvic drop and truncal lean towards the shorter side [11]. Although these alterations suggest a potential mechanism for increased pull on the greater trochanter of the longer side, it is currently unclear whether there is an association between LLI and prevalent GTPS. There is a need for research to test this widely held assumption. Our study therefore assessed the association between LLI and GTPS after adjusting for potential covariates.

## Materials and methods

### Participants

The Multicenter Osteoarthritis Study (MOST) is a cohort of 3,026 adults aged 50 to 79 years recruited from the community because they either had symptomatic knee osteoarthritis or were at high risk of knee osteoarthritis based on obesity, knee pain, or previous knee injury. Recruitment for MOST has been described elsewhere [16]. Our study was a cross-sectional analysis of the initial visit of the 1,519 subjects at one MOST clinical site. Institutional Review Board approval was obtained at each of the investigators' institutions prior to initiating recruitment and research protocols. The protocol was in compliance with the Declaration of Helsinki and all subjects provided written informed consent prior to participation.

### Definition of greater trochanteric pain syndrome

GTPS was defined as tenderness over a greater trochanter on physical examination of subjects who reported pain over the lateral hip on a pain diagram. Subjects with generalized myofascial tenderness to palpation (defined below) were not included in the definition of GTPS. Physical examination was performed on the subset of subjects who complained of pain, aching, or stiffness in either hip or knee on most days over the past month. Subjects who did not complain of knee or hip pain were considered not to have GTPS, as the absence of pain in the region or radiating distally from this area would preclude the presence of a pain syndrome. Since we wished to study trochanteric area pain, not referred pain from the hip joint, subjects with suspected hip osteoarthritis based on hip internal rotation ≤ 15° in the context of pain with internal rotation were excluded from the definition of GTPS [17,18].

### Clinical examinations

Physical examination included asking subjects 'is this tender or painful' while applying 1.4 to 3.0 kg fingertip pressure over the lateral and posterior aspects of each greater trochanter with the subject in the lateral decubitus position [19]. Generalized myofascial tenderness was defined by an affirmative response to the same question when 1.4 kg pressure was applied over the soft tissue 2 cm proximal to the medial joint line of the knee as well as at two or more of the following points: left and right proximal trapezius, and left and right extensor mass immediately distal to the lateral epicondyle of an elbow [20]. Trained examiners, certified in the standardized protocol, calibrated the amount of physical pressure they would use during the examination at the start of each session, and underwent annual recertification to assure uniformity in following the examination protocol. Examiners used a Chatillon CMD 10-1 dolorimeter (Ametek US Gauge Division, Largo, FL, USA) daily to calibrate finger pressure [21,22] to 1.4 and 3.0 kg pressure, prior to palpating the subjects' greater trochanters [23,24].

### Leg-length assessment

Weight-bearing anteroposterior full-limb radiographs were completed on all subjects. Radiographs were read by trained technologists who measured the leg length, defined as the distance from the femoral head center to the tibial mid-plafond point. The femoral head center was marked using concentric circles to outline the contour of the femoral head and to identify the center point. The mid-plafond point is the point in the distal tibia directly over the center of the talar dome. Three additional observers then completed radiographic leg-length measurements, and any discrepancies were adjudicated to ensure consensus on all measurements.

### Statistical analyses

We assessed the association between LLI and side-specific GTPS after adjusting for leg length, age, and sex using logistic regression analyses and generalized estimating equations to adjust for the correlation between hips [25]. For categorical analysis, a clinically significant LLI was defined at a threshold ≥ 1 cm; this defined threshold identified LLI regardless of whether the affected limb was longer or shorter. Categorical variables therefore distinguished whether the limb ipsilateral to the GTPS was shorter than, longer than, or equal in length to (<1 cm difference) the contralateral lower limb. To avoid missing a smaller LLI that could be a significant predictor of GTPS, we also analyzed LLI as a continuous variable. For this analysis, the leg length contralateral to the painful trochanter was subtracted from the ipsilateral leg length, yielding a positive LLI when the ipsilateral lower limb was longer and a negative LLI when the ipsilateral lower limb was shorter.

To assess the independent effects of leg length, LLI, and whether the longer lower limb was ipsilateral or contralateral to the GTPS diagnosis, regression models were tested: with leg length, ipsilateral long limb, ipsilateral short limb, age, and sex; with leg length, LLI, age, and sex; and with ipsilateral long limb, ipsilateral short limb, age, and sex.

To confirm that the definition of GTPS was sufficiently sensitive, the above analyses were repeated using a broader definition for GTPS, including all subjects reporting hip *or knee *pain, who had greater trochanteric tenderness to palpation (excluding those with generalized myofascial tenderness).

## Results

### Participants

**Figure 1:**
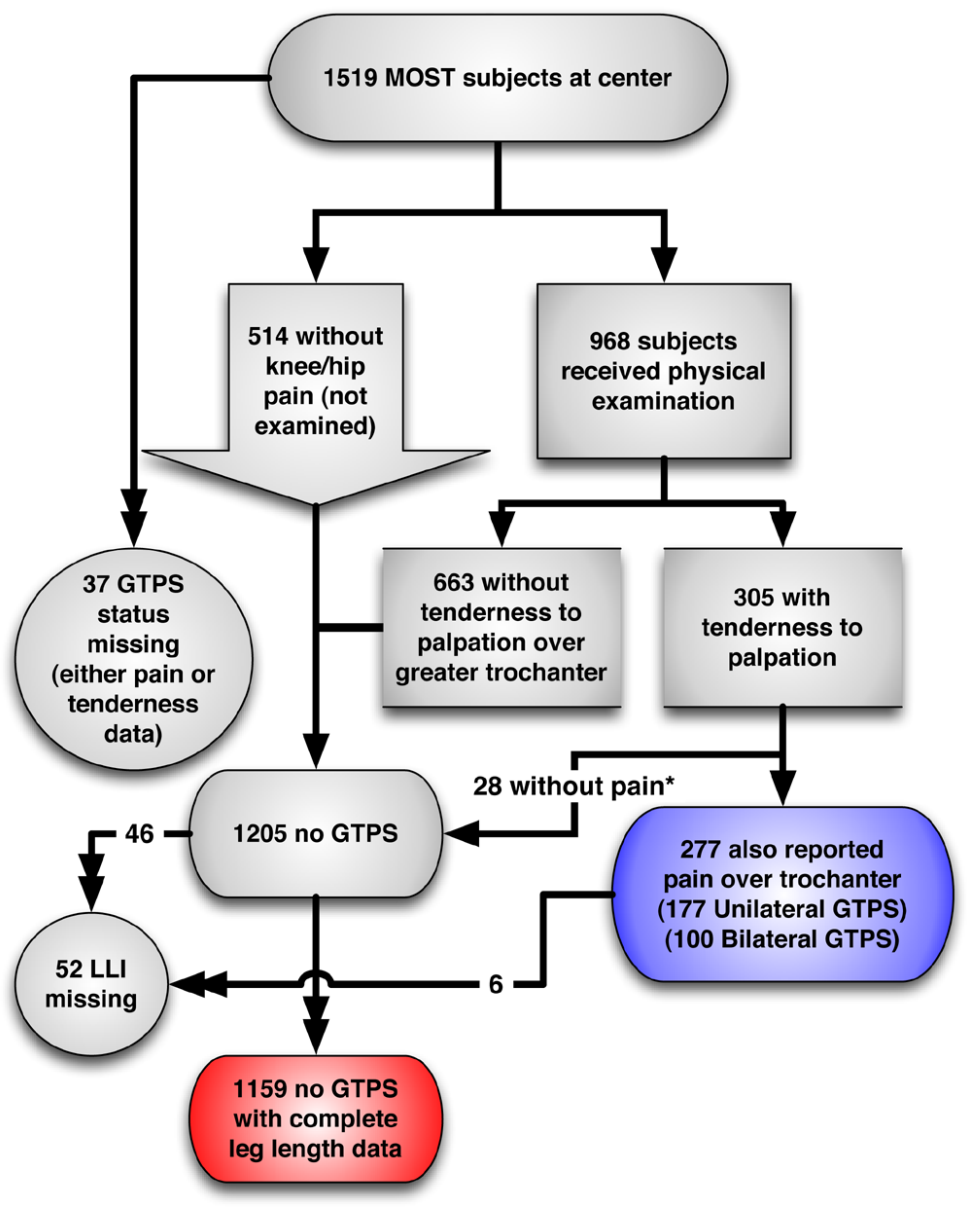
Subject recruitment and group disposition. *Did not meet definition for greater trochanteric pain syndrome (GTPS). LLI, leg-length inequality; MOST, Multicenter Osteoarthritis Study.

A total of 1,482 subjects (2,877 lower limbs) were eligible for analysis of GTPS. Leg-length information was missing for 52 subjects, leaving 1,430 subjects eligible for the analysis of GTPS and leg length (Figure 1). The included subjects' mean ± standard deviation age was 62.4 ± 8.2 years, and 59.8% were female. A total of 372 lower limbs from 271 subjects (19.0%) met the definition for having GTPS (12.2% of persons in the cohort had unilateral GTPS, and an additional 6.8% had bilateral GTPS).

### Leg-length inequality

The mean ± standard deviation leg length was 86.4 ± 7.1 cm with a range of 67.7 to 169.5 cm. The LLI ranged from 0 to 6.4 cm. A categorical LLI (difference ≥ 1 cm) was present in 37 subjects with GTPS (nine cases with ipsilateral shorter leg, 11 cases with ipsilateral longer leg, and 17 cases with bilateral GTPS) and in 163 subjects without GTPS; therefore, 13.7% of subjects with GTPS had a LLI compared with 14.1% of those without GTPS (*P *= 0.86 by Fisher's exact test). Adjusting for age and sex, LLI was not a significant predictor of hip-specific GTPS status, with an odds ratio of 1.17 (95% confidence interval = 0.79 to 1.73).

Additionally, treating LLI as a continuous variable and adjusting for age and sex, LLI was not associated with GTPS. Separate regression analyses (1) without including leg length, (2) with indicator variables for an ipsilateral longer lower limb, and (3) with indicator variables for an ipsilateral shorter lower limb also did not reveal LLI to be a statistically significant predictor of GTPS status (all *P *> 0.2). Female sex was significantly associated with the presence of GTPS with an adjusted odds ratio of 3.04 (95% confidence interval = 2.07 to 4.47), however, after controlling for LLI, leg length, and age.

Broadening the definition of GTPS from subjects with only lateral hip pain to include all subjects with hip or knee pain complaints who also had greater trochanteric tenderness to palpation (still excluding those with generalized myofascial tenderness) also did not reveal any statistically significant associations with LLI in either continuous or categorical analyses.

## Discussion

In the present study involving 2,877 lower limbs (1,430 subjects with no missing data), no association was found between GTPS and LLI. These conclusions were robust to use of various definitions for LLI.

The most probable explanation for this finding is that, despite opinion found in textbooks, no association actually exists. This reasoning is consistent with the near absence of data supporting an association between GTPS and LLI. The only published evidence of such an association we found was a single case series of four subjects [6]. Alternatively, insufficient power could be a reason for not finding an association. The relatively large number of cases in our study (*n* = 271) suggests that a clinically significant association would not have been missed. A larger sample size, however, may enable detection of smaller differences.

LLI has been measured in many ways. Radiography has been considered the gold standard for measuring LLI, with several accepted methods, including full-limb X-rays, scanograms (three separate exposures of the hip, knee, and ankle), and computerized digital X-ray scans [26]. Magnetic resonance imaging and computed tomography can also be used, but these are costly and cumbersome approaches. In addition, magnetic resonance imaging or computed tomography are completed in the supine position and may be subject to error relating to leg-length change when not weight-bearing – for example, a unilateral pes planus, varus, or valgus knee deformity that may increase in a weight-bearing position. Although it is subject to parallax error, the full-limb weight-bearing X-ray method, with measurement from the femoral head to the ankle, is the most commonly used technique currently – and the method is reliable [27]. The measurement of LLI by full-limb radiography in our study should not be a significant source of error and would not explain the null findings.

Diagnosis of GTPS in the present study was based on subjects' report of pain as well as on a standardized physical examination conducted by trained research nurses. Repetition of this measure on more than 1,000 subjects by a small number of research nurses, who were trained and certified in the protocol, and who calibrated their fingertip pressure daily, minimized variability in the measure.

The prevalence of LLI in our subjects was 14.3% when a cutoff ≥ 1 cm was used, and would have been 11.1% if LLI was defined as >1 cm. Both of these estimates are comparable with the range of LLI prevalence reported in other studies (4% to 22%) [14,28]. Differences from the study reporting a lower prevalence (4%) may be due to different methods or subject characteristics [28]. That report used ultrasonography to measure leg length from the femoral head to the floor. Imaging methods have been shown to be significantly less variable than tape-measure methods, but also less convenient for routine clinical use [28-31]. Additionally, the lower-prevalence study enrolled subjects with a mean age of 33 years and no history of lower limb abnormalities [28]. Those subjects probably differed from our subjects in the MOST cohort, who were over age 50 years and had a high prevalence of knee pain, injury, or surgery. Comparison with another report of LLI prevalence is difficult due to methodological differences (a retrospective review of records of patients who were prescribed orthopedic devices) [32].

The increased odds for GTPS in women were consistent with prior reports [4,33]. The mechanism for increased odds for GTPS in women, however, is unclear [34]. This association could relate to anatomy (such as the flared pelvic rim in women altering the pull of the iliotibial band), physiology (hormonal effects on bursal irritation or pain generators), or differences in activity between men and women.

Despite some limitations, the use of commonly available clinical methods and findings consistent with prior research results lends strength to the findings of the present study. From a therapeutic perspective, although there appears to be no association between GTPS and LLI, this would not necessarily indicate that use of a heel lift would be unhelpful for other lower limb symptoms. In particular, patients with a LLI of 2.5 to 3.5 cm have been reported to have greater ground reaction forces [8], quadriceps activity [9], and hip joint forces [10]. This area of heel lift usage requires further research, and we anticipate that if a shoe insert were found to benefit patients with GTPS, it would probably act through factors other than correction of LLI.

## Conclusion

The present study found no evidence to support an association between LLI and GTPS. This advanced understanding will enable improved accuracy in future textbooks as well as inform future research.

## Abbreviations

GTPS = greater trochanteric pain syndrome; LLI = leg-length inequality; MOST = Multicenter Osteoarthritis Study.

## Competing interests

The authors declare that they have no competing interests.

## Authors' contributions

NAS developed the study hypotheses and analysis plan, participated in the statistical analysis, and drafted the manuscript. WH participated in the data collection and manuscript preparation. DTF participated in the study design and manuscript preparation. MY conducted the statistical analysis and participated in manuscript preparation. JCT participated in the study design, analysis, and interpretation and in manuscript preparation. JRC and MCN participated in the manuscript review. All authors read and approved the final manuscript.
